# Network analyses predict major regulators of resistance to early blight disease complex in tomato

**DOI:** 10.1186/s12870-024-05366-0

**Published:** 2024-07-06

**Authors:** Christopher S. Tominello-Ramirez, Lina Muñoz Hoyos, Mhaned Oubounyt, Remco Stam

**Affiliations:** 1 Department of Phytopathology and Crop Protection, Institute for Phytopathology, Christian Albrechts University, Kiel, Germany; 2 Phytopathology, TUM School of Life Sciences Weihenstephan, Technical University of Munich, Freising, Germany; 3 Institute for Computational Systems Biology, University of Hamburg, Hamburg, Germany

**Keywords:** Comparative transcriptomics, Plant immunity, *Alternaria*, Early blight, Brown leaf spot, Tomato, Co-expression network, Gene regulatory network

## Abstract

**Background:**

Early blight and brown leaf spot are often cited as the most problematic pathogens of tomato in many agricultural regions. Their causal agents are *Alternaria* spp., a genus of Ascomycota containing numerous necrotrophic pathogens. Breeding programs have yielded quantitatively resistant commercial cultivars, but fungicide application remains necessary to mitigate the yield losses. A major hindrance to resistance breeding is the complexity of the genetic determinants of resistance and susceptibility. In the absence of sufficiently resistant germplasm, we sequenced the transcriptomes of Heinz 1706 tomatoes treated with strongly virulent and weakly virulent isolates of *Alternaria* spp. 3 h post infection. We expanded existing functional gene annotations in tomato and using network statistics, we analyzed the transcriptional modules associated with defense and susceptibility.

**Results:**

The induced responses are very distinct. The weakly virulent isolate induced a defense response of calcium-signaling, hormone responses, and transcription factors. These defense-associated processes were found in a single transcriptional module alongside secondary metabolite biosynthesis genes, and other defense responses. Co-expression and gene regulatory networks independently predicted several D clade ethylene response factors to be early regulators of the defense transcriptional module, as well as other transcription factors both known and novel in pathogen defense, including several JA-associated genes. In contrast, the strongly virulent isolate elicited a much weaker response, and a separate transcriptional module bereft of hormone signaling.

**Conclusions:**

Our findings have predicted major defense regulators and several targets for downstream functional analyses. Combined with our improved gene functional annotation, they suggest that defense is achieved through induction of *Alternaria*-specific immune pathways, and susceptibility is mediated by modulating hormone responses. The implication of multiple specific clade D ethylene response factors and upregulation of JA-associated genes suggests that host defense in this pathosystem involves ethylene response factors to modulate jasmonic acid signaling.

**Supplementary Information:**

The online version contains supplementary material available at 10.1186/s12870-024-05366-0.

## Background

Tomato (*Solanum lycopersicum L.*) yields are at significant risk from all forms of biotic stress, including over 200 diseases [1]. *Alternaria* spp. are the causal agents of early blight (EB) and brown leaf spot (BLS), often cited as the most important foliar pathogens of tomato [2, 3]. EB of tomato is caused by large-spored *Alternaria* sect. *Porri*, specifically *Alternaria linariae* (Neerg.) E.G.Simmons, commonly synonymized as *Alternaria tomatophila* E.G.Simmons, or the potato pathogen *Alternaria solani* Sorauer [4]. BLS is caused by small-spored *Alternaria* sect. *Alternaria*, specifically *Alternaria alternata* (Fr.) Keissl., a taxonomic concept that encompasses numerous *formae speciales* and is known to infect over 100 host species [5]. EB and BLS are a symptomatology continuum of small dark-brown lesions developing on leaves, stems, and fruits that develop characteristic concentric rings, about 5 mm in diameter for BLS and about 15 mm in diameter for EB, culminating in complete defoliation of the plant if left unmanaged [6]. Both diseases will be referred to hereafter as early blight/brown leaf spot disease complex (EBDC) because of the significant overlap in pathogenesis [7].

Fungicide remains an effective preventative measure against EBDC [8], but despite reproducing clonally, *A. linariae* and *A. alternata* are considered medium-risk and high-risk pathogens respectively in developing fungicide resistance to several chemistries [9]. Due to the environmental, financial, and biosecurity-related cost of intensive fungicide use, identification of genetic resistance mechanisms to EBDC remains an important albeit elusive target. Resistance is predicted to proceed quantitatively, polygenically, and inherited recessively due to the necrotrophic lifestyle of its causal agents [10, 11]. Accordingly, only polygenic and quantitative EBDC resistance mechanisms have been identified in tomato [8].

Genetic defense responses in plants are mediated by large-scale shifts in gene expression [12], secondary metabolism [13, 14], and tailored to specific pathogen lifestyles using phytohormones. Salicylic acid (SA) is the major phytohormone that regulates resistance against biotrophs, and jasmonic acid (JA) for necrotrophs [11]. SA promotes defense towards biotrophs at the expense of susceptibility towards necrotrophs by antagonizing JA signaling via a myriad of mechanisms [15]. Other than SA and JA, all other phytohormones participate in plant immunity by balancing the hormonal crosstalk [16]. Notably, ethylene (ET) can abolish SA primacy during SA/JA antagonism, acting as a switch between these two distinct immunity modalities, and highlights its importance during necrotroph defense [17]. This simple paradigm predicts that SA should promote EBDC infection, and JA/ET should suppress it. Indeed, foliar application of methyl jasmonate methyl ester can reduce EBDC symptoms [18], yet foliar application of SA has been shown to reduce the occurrence of lesions caused by EBDC [19]. Priming tomato plants with SA induces PATHOGENESIS-RELATED (PR) proteins [20], a diverse gene assemblage shown to be upregulated in response to EBDC [21]. A PR protein-induced hypersensitive response (HR) may play a role in mediating defense in EBDC-resistant tomato breeding lines, with the hypothesis that a functional difference between toxin-induced cell death and the programmed cell death (PCD) of HR underlies the resistance [22]. Furthermore, host colonization often fails to advance beyond tomato cells exhibiting EBDC-induced HR, an SA-regulated process, and highlights the importance of early immune events [23]. Although HR is known to occur alongside other defense responses such as callose deposition and secretion of antimicrobial metabolites [24], it remains the case that unlike other necrotrophic pathosystems, HR is not determinative of susceptibility to EBDC in tomatoes. While the role of HR, and possibly SA, is somewhat enigmatic in EBDC pathogenesis [25], the importance of JA signaling has been demonstrated in the EBDC/potato pathosystem [26, 27]. Clarity of the immune response mechanisms remains insufficient in this complex pathosystem, especially in tomato which lacks a comparative study of the genetic responses between EBDC resistance and susceptibility.

To begin disentangling the molecular participants of the early events in EBDC resistance in tomato, we sequenced transcriptomes of Heinz 1706 tomatoes 3 h post infection (hpi) with EBDC. In the absence of strongly EBDC-resistant tomato germplasm, we treated Heinz 1706 tomatoes with the small-spored EBDC isolate CS046, collected in situ from wild tomatoes in Peru and shown to have little virulence on Heinz 1706 tomato [28, 29]. These transcriptomes are compared with three other treatments, the highly virulent large-spored EBDC isolate 1117–1, collected from infected tomatoes in Germany [28, 29], chitin as a general elicitor for plant microbe responses, and a mock treatment of water. It has been shown that avirulent isolates of *Zymoseptoria tritici* induce more defense genes, and at earlier time points than virulent isolates, highlighting the potential of early time points with weakly virulent pathogens to detect novel defense genes [30]. We have previously shown that elicitor treatment of tomato relatives leads to strong defense induction visible at the transcriptomic and plant hormone level [31]. We also showed that dozens of pathogen-induced differentially-abundant metabolites can be identified between CS046- and 1117–1-treated tomatoes at 3 hpi, including antifungal compounds [29]. To identify a defense-related module of gene expression amongst the diverse genes regulated by the hormone flux, we constructed a weighted gene co-expression network and identified a subnetwork associated with EBDC defense. To identify the genes with the largest influence on the defense subnetwork topology, we identified hub genes with high eigenvector centrality and constructed a directed gene regulatory network (GRN). From these genes, we identified novel genes with roles in EBDC defense.

## Methods

### Sample growth conditions, experimental treatment, and sample collection

Our Heinz 1706 tomatoes were grown in a phytotron-type walk-in growth chamber at 23 °C with a 12-h photoperiod. All treatments were performed on 3-week-old meristem cuttings propagated from 6 week old seedlings. The fungal isolates CS046 and 1117–1 were grown in a Sanyo MLR-351H Versatile Environmental Test Chamber (Moriguchi, Japan) growth cabinet at 23 °C, 60% relative humidity, and a 24-h photoperiod in both the visible and ultraviolet range. Fungi were propagated on synthetic nutrient agar medium [32] in standard 100 mm petri plates. Conidia were harvested from plates approximately one month after inoculation by flooding the plate with sterile millipore-filtered water. The yield of conidia was quantified using a haemocytometer, and diluted to achieve 3 × 10^4^ conidia × 1 mL^−1^ for plant infection. Crab shell chitin was prepared by freezing it in liquid nitrogen and grinding it with a mortar and pestle until a very fine powder was achieved. We spray-infected the above-ground tissues of the tomatoes with the conidia suspension until inoculum runoff was achieved, indicating droplet saturation. The same treatment method was used for 50 µg chitin × 1 mL^−1^, and with sterile millipore-filtered water. Each treatment had 4 biological replicates. Plants were placed in a plastic tub with a lid on to ensure 100% relative humidity, as measured with an AHT20 temperature and humidity sensor (Adafruit, New York City, NY, USA). We collected treated leaves after 3 h incubation, wrapped them in aluminum foil, flash-froze them in liquid nitrogen, and stored them at -80 °C prior to RNA extraction. To verify near-ubiquitous conidium germination for both isolates at 3 hpi, we drop-infected leaves of Heinz 1706 tomatoes grown in the research greenhouse at the University of Kiel with 10µL of 3 × 10^4^ conidia 1 mL^−1^ of both CS046 and 1117–1, and used a light microscope to confirm the presence of germ tubes.

### RNA extraction, library construction, and read mapping

We extracted total RNA using RNeasy Plant kits (Qiagen, Venlo, Netherlands). The mRNA library for 3’ sequencing was generated using the QuantSeq 3’mRNA-Seq Library Prep Kit (Lexogen, Vienna, Austria) and then sequenced using an HiSeq2500 (Illumina, San Diego, CA, USA) with Rapid SBS v2 chemistry to generate 100 bp single-end reads. For all RNA extraction, quality assessment methods, mRNA library generation, and sequencing utilized the manufacturer’s recommended methodologies unchanged. We used ‘Trimmomatic’ [33] for initial quality filtering and adapter trimming of the raw sequencing reads, aligned trimmed RNA-seq reads to the International Tomato Annotation Group (ITAG) genome version 4 [34] using ‘HISAT2’ [35], and quantified aligned sequencing reads using ‘featureCounts’ [36]. All software used the default settings except the following Trimmomatic settings: LEADING:3, TRAILING:3, SLIDINGWINDOW:4:15, and MINLEN:40.

### Differential gene expression analysis

We used the R package ‘DESeq2’ ver. 1.38.3 [37] to perform the differential gene expression analysis. The transcript counts matrix was pre-filtered for genes with less than 10 total normalized read counts across all samples prior to differential expression analysis. Log_2_ fold change (LFC) values were shrunken using the ‘apeglm’ method [38] to reduce noise [37], and *p*-values were corrected using the Benjamini–Hochberg method to calculate their false discovery rates (FDR) [39]. We considered genes to be differentially expressed if their |LFC|> 1 and FDR < 0.05. All subsequent analyses utilized the normalized gene counts matrix with a regularized log_2-_transformation to reduce heteroscedasticity. The upset plot was generated using the R package ‘ComplexHeatmap’ ver. 2.14.0 [40], and the PCA was generated using DESeq2.

### GO term enrichment

We used the Cytoscape ver. 3.10.1 [41] app BiNGO ver. 3.0.5 [42] for GO term enrichment and visualization using the hypergeometric statistical test and FDR correction. GO term annotations were acquired primarily through ITAG resources. Any remaining genes without GO terms were annotated using PANNZER2 ver. 15.12.2020 [43] with default settings, and filtered for hits with a positive predictive value (PPV) above 0.4 [44].

### Analysis of canonical immunity elements

Receptor-like kinases (RLKs) were identified using ‘DeepLRR’ ver. 1.01 [45] searching for the ‘LRR_RLK’ protein type, and appended this list with RLK genes from Sakamoto et al*.* [46]. Hits were filtered for subcellular location type ‘Cell membrane’ using the ‘DeepLoc’ ver. 2.0 web service using high-throughput parameters [47], tested for transmembrane domains using the ‘deepTMHMM’ web service [48], and ligand-binding domains and kinase domains were identified using HMMER ver. 3.3.2 [49] and the ‘CD-search’ ver. 3.20 web service [50]. Receptor-like proteins (RLPs) were identified in a similar manner by using the ‘LRR_RLP’ protein type option of DeepLRR and appending this lists with RLP genes from Kang and Yeom [51], hits were filtered for the ‘Cell membrane’ subcellular location using DeepLoc, transmembrane domains identified using deepTMHMM, GPI-anchor domains identified using the ‘NetGPI’ ver. 1.1 web service [52], and ligand-binding domains classified using HMMER and CD-search. Receptor-like cytoplasmic kinases (RLCKs) were identified by filtering the list of RLCKs from Sakamoto et al*.* [46] for subcellular location ‘cytoplasm’ using DeepLoc and the ligand-binding and kinase domains analyzed using HMMER and CD-search. Nucleotide-binding domain and leucine-rich repeat proteins (NLRs) were identified by filtering HMMER hits for ‘NB-ARC’, and examining the leucine-rich repeat (LRR) domains and N-terminal domains for completeness and structure using CD-search. To identify further canonical immunity genes, HMMER was used to conduct an ‘hmmscan’ on the ITAG4.0 tomato genome [34]. Calcium-responsive proteins and respirator burst oxidase homolog (Rboh) proteins were identified by filtering HMMER hits for ‘EF-hand’ and ‘Ferric_reduct’, respectively, and using CD-search to analyze the protein domains. Mitogen-activated protein kinases (MAPKs) were referenced from the list provided by Wu et al*.* [53]. Transcription factors (TFs) were referenced by the list provided by PlantTFDB5.0 [54]. Auxin responsive genes were identified by filtering HMMER hits for ‘f-box’, ‘AUX_IAA’, ‘Auxin_resp’, ‘Auxin_inducible’, ‘GH3’, ‘Mem_trans’, and ‘Aa_trans’ to identify Transport Inhibitor Response 1/Auxin Signaling F-Box, Aux/IAA, auxin response factors, small auxin up RNAs, GH3, PIN, and AUX/LAX proteins, respectively. Abscisic acid (ABA) responsive genes were identified by filtering HMMER hits for ‘Polyketide_cyc2’, ‘Pkinase’, ‘bZIP’, ‘PP2C’, and ‘SLAC1’ to identify PYR/PYL/RCAR ABA receptors, SnRK2 ABA-activated protein kinases, ABF/AREB/ABI5 TFs, ABI1/ABI2 proteins, and SLAC1/SLAH ion channels, respectively. Cytokinin responsive genes were identified by filtering HMMER hits for ‘Response_reg’ and ‘Hpt’ to identify AHK2-4/CRE1 histidine kinase receptors and HPt histidine phosphotransfer proteins. ET responsive genes were identified by filtering HMMER hits for ‘Gamma-thionin’ to identify defensins, referencing the list provided by Liu et al*.* [55] to identify ET receptors, constitutive triple response, ET insensitive 2 (EIN2), EIN3, EIN3-like, EIN3 binding F-box, 1-Aminocyclopropanecarboxylic acid (ACC) synthase, and ACC oxidase proteins, and by filtering the PlantTFDB5.0 TF list for ‘ERF’ to identify ethylene response factors [54]. Gibberellic acid (GA) responsive genes were identified by filtering HMMER hits for ‘Hormone_Rec’, ‘GRAS’ and ‘f-box’ to identify gibberellin insensitive dwarf1 GA receptors, DELLA proteins, and SLY1/SNE proteins, respectively. Jasmonic acid (JA) responsive genes were identified by filtering HMMER hits for ‘JAZ’, bHLH-MYC_N’, ‘NINJA’, and ‘LisH_TPL’ to identify jasmonate ZIM-domain proteins, MYC TFs, novel interactor of JAZ proteins, and TOPLESS/TOPLESS-related genes, respectively. The canonical coronatine insensitive 1 gene was identified using NCBI resources. Salicylic acid (SA) responsive genes were identified by filtering HMMER hits for ‘BTB’, ‘DOG1’, ‘Chorismate_bind’, and ‘Patatin’ to identify non-expressor of PR genes (NPR) 1–3 proteins, isochorismate synthase 1, and enhanced disease susceptibility 1 proteins, respectively. Brassinosteroid responsive genes were identified by filtering HMMER hits for ‘bHLH-MYC_N domain’ and ‘14–3-3’ to identify brassinazole-resistant1 and 14–3-3 proteins respectively. Pathogenesis-related genes were considered true PR genes if they were i) identified as orthologous using ‘orthofinder’ software ver. 2.5.5 [56], ii) shared all conserved domains using CD-search, iii) had their best NCBI BLAST [57] hits against the ‘ref_seq’ database as the originally published PR gene [20], and were iv) within the same genetic clade as their confirmed orthologs in other taxa in a fastest minimum evolution phylogenetic tree using NCBI COBALT [58]. Genes were plotted using the R package ‘ggplot2’ ver. 3.4.2 [59].

### Weighted gene co-expression network analysis

We used the R package ‘WGCNA’ ver. 1.72–1 [60] to construct a weighted gene co-expression network. The input matrix for network generation was variance stabilized and filtered for low-expression genes by determining the threshold when the normalized counts increase exponentially from the median value as calculated using the R package ‘segmented’ (mean normalized expression < 3.501). The input matrix was similarly filtered for genes with low variance (variance < 0.027). After appending our list of high-variance and high-expression genes with the remaining differentially expressed genes (DEGs) that were filtered out, the gene expression of each sample was correlated to its experimental treatment class, a binary trait, and therefore a signed network was constructed using Pearson’s correlations as recommended by the author of WGCNA. All other network parameters were set to default values except the following parameters: ‘deepSplit’ set to 0 to suppress module splitting, minimum module size set to 30, and ‘mergeCutHeight’ set to 0.37 to prevent insignificantly-correlated modules of the minimum size from overcoming the module merger threshold. The network was then filtered for low edge weight (edge weight < 0.154) using the R package ‘segmented’ before visualization with Cytoscape. Hub genes were identified by calculating eigenvector centrality of individual co-expression modules using the R package ‘igraph’ ver. 1.5.0 [61]. Heatmaps were generated using the R package ‘ComplexHeatmap’.

### Gene regulatory network

After obtaining a comprehensive list of tomato TFs from PlantTFDB5.0 [54], we used the R package ‘Genie3’ ver. 1.20.0 [62] to infer a directed GRN of the entire input matrix of genes filtered for low-variance and low-expression. The resulting GRN was visualized using putative regulatory links filtered out based on low model fit and visualized using Cytoscape. GRN hub genes were identified by calculating eigenvector centrality of the entire GRN using the R package ‘igraph’. Heatmaps were generated using the R package ‘ComplexHeatmap’.

## Results

### Early responses to chitin and conidiospores of CS046 and 1117–1 each induce distinct transcriptome responses

To investigate the early defense responses to EBDC, we generated an RNA-seq dataset from Heinz 1706 tomato plants treated with the weakly virulent isolate CS046 (*A. alternata*), the strongly virulent isolate 1117–1 (*Alternaria* sect. *Porri*), and chitin for 3 h. To validate that both *Alternaria* isolates would in principle be able to infect and to eliminate, for example, differences in germination rate as a significant source of error, we re-isolated droplets with spores of both isolates from Heinz 1706 leaves 3 h after infection. Microscopic analysis confirms that both isolates can germinate on the leaves (Fig. S1a-b). Inspection of the leaves 72 h after inoculation confirms the observations by Muñoz Hoyos 2023, that 1117–1 is virulent, and CS046 has little to no virulence on Heinz 1706 tomato leaves (Fig. S1c), thus indicating that a successful defense response must be activated 3 hpi in CS046-treated plants.

**Fig. 1:**
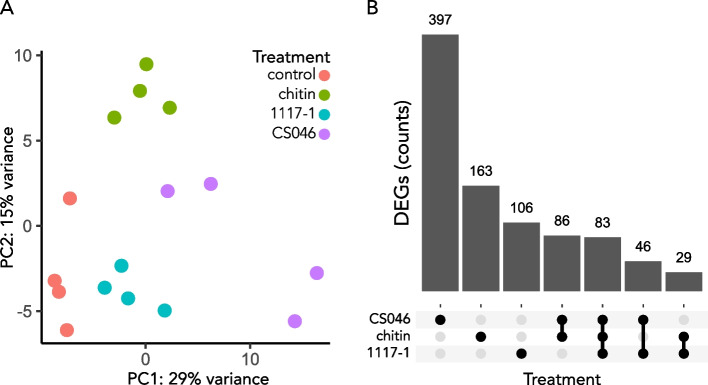
All treatments induce distinct, mostly unique transcriptome responses. **A** Principal component analysis plot to visualize the distribution and clustering of samples based on their gene expression. Dots represent an individual sample replicate. Dot color represents treatment class. The axes represent the variance explained by the first two principal components. **B** Upset plot showing the intersections and unique sets of differentially expressed genes (DEGs) amongst the treatments. The x-axis shows the categories of the comparison sets, and the y-axis represents the number of DEGs within a comparison set. The horizontal bars represent the individual treatments, the black dots indicate which treatments are compared, and the vertical lines represent the intersection of treatments for a given comparison. The numbers above the vertical bars indicate the number of DEGs in a comparison set

Our yield of sequencing reads was 7.6 million reads per sample with a mapping rate of 86%, and identified 910 DEGs across all treatments. Principal component analysis shows discrete and distinct responses for all treatments, with sample replicates segregating together by gene expression patterns (Fig. 1a). An upset plot shows that the treatment with the strongest response is CS046-treated tomatoes with 397 unique DEGs, and the weakest response is the 1117–1 treatment with 106 unique DEGs (Fig. 1b). 83 DEGs (9%) were shared between all treatments; in contrast, 641 DEGs (72%) were unique to the specific treatments (Fig. 1b).

### Gene expression for the CS046 treatment is more enriched for defense-related gene ontologies than for the 1117–1 treatment

To investigate the biological activities of the transcriptional responses to the treatments, we performed GO term enrichment analysis on the sets of DEGs of each individual treatment. The transcription response to chitin, 1117–1, and CS046 treatment yielded 112, 135, and 175 total enriched GO terms respectively (Table S1), indicating stronger coordination of the early defense response to the weakly virulent EBDC isolate. The two EBDC isolates elicit similar types of defense responses, although conspicuously missing from the 1117–1 treatment is cell wall remodeling (Table 1). The most notable difference between the fungal treatments and the chitin treatment is the expanded enrichment of GO terms for secondary metabolite biosynthesis (Table 1). All treatments share a common response in biological processes for ROS activities, secondary metabolite biosynthesis, stress responses, and hormone responses; molecular functions for catalytic activity, transferase activity, and molecule binding; and cellular compartments for extracellular region, plasma membrane, and the chloroplast (Table 1). Considering the greater gene induction in the CS046 treatment (Fig. 1) and the observation that TF activity is an enriched GO term, CS046 has a greater induction in the number of genes for each of these gene categories, despite having similar gene types in the 1117–1 treatment.

**Table 1:** Summary of gene ontology enrichment for each experimental treatment class and gene ontology class

	**Biological Process**		**Molecular Function**		**Cellular Compartment**	
	**GO description**	**no. enriched GO terms**	**GO description**	**no. enriched GO terms**	**GO description**	**no. enriched GO terms**
chitin	cell wall remodeling	18	catalytic activity	12	plasma membrane	9
	ROS activities	10	molecule binding	12	chloroplast	7
	stress response	7	transferase activity	2	extracellular region	5
	SMB	5	hydrolase activity	2		
	hormone response	3				
1117–1 *(Alternaria* sect.* Porri)*	SMB	28	molecule binding	17	plasma membrane	7
	stress response	15	catalytic activity	14	chloroplast	7
	hormone response	13	transferase activity	3	extracellular region	2
	primary metabolism	11	enzyme inhibition	4		
	ROS activities	10	antioxidant activity	1		
CS046* (Alternaria alternata)*	SMB	29	catalytic activity	17	plasma membrane	9
	hormone response	19	molecule binding	16	chloroplast	9
	stress response	19	transferase activity	11	extracellular region	4
	cell wall remodeling	19	enzyme inhibition	1		
	ROS activities	5	transcription factor activity	1		

*GO* gene ontology, *ROS* reactive oxygen species, *SMB* secondary metabolite biosynthesis

### Canonical plant immunity components suggest the importance of phytohormone signaling in early response to EBDC

**Fig. 2:**
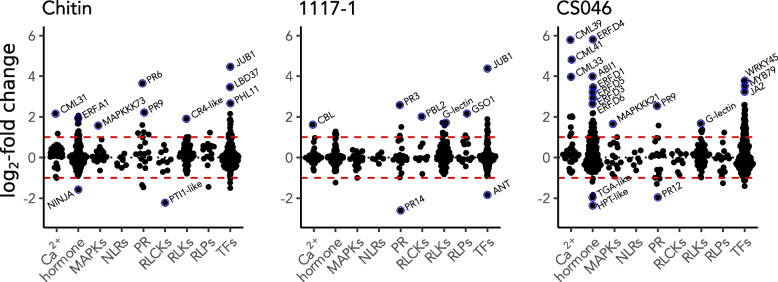
The early defense response to EBDC involves gene transcription, Ca2 + signaling, and hormone signaling. Expression patterns of tomato genes with significant differential expression (FDR < 0.05) in canonical immune signaling pathways after treatment with chitin or spores of either a virulent (1117–1) or avirulent (CS046) isolate of EBDC. The black dotted line is set to the up/down-regulation inflection point (LFC = 0); the red dashed line is set at the LFC threshold to be considered a DEG (LFC =|1|); blue circles and labels annotate notable genes with high differential expression

To interpret the immune signaling events during EBDC defense, we investigated the activation of canonical pattern-triggered-/effector-triggered immunity (PTI/ETI) pathways [63]; calcium signaling, hormone responses, MAP kinases, NLRs, PRs, RLPs, RLKs, RLCKs, and TFs. We found 438 genes with significant differential expression in these categories and 136 DEGs which also satisfy the log_2_ fold change threshold (Table S2). *MAPKs*, NLRs, *PRs,* RLCKs, RLKs, and RLPs only account for 32 DEGs, while the remaining 104 DEGs are split between calcium signaling, hormone responses, and TFs, indicating the importance of these three gene categories (Fig. 2). Tomatoes treated with CS046 have the strongest immune response with 73 DEGs identified as canonical immune system components. Tomatoes treated with chitin and 1117–1 yielded 41 and 28 DEGs respectively, consistent with the global transcription responses of the individual treatments (Fig. 1). Notably, some of the most strongly induced genes from the CS046 treatment are five *ETHYLENE RESPONSE FACTORs* (*ERFs*) from the D clade [64], which have not been previously identified with a role in EBDC defense.

### Defense-related co-expression network characterized by hormone signaling, cellular stress responses, and secondary metabolite biosynthesis

**Fig. 3:**
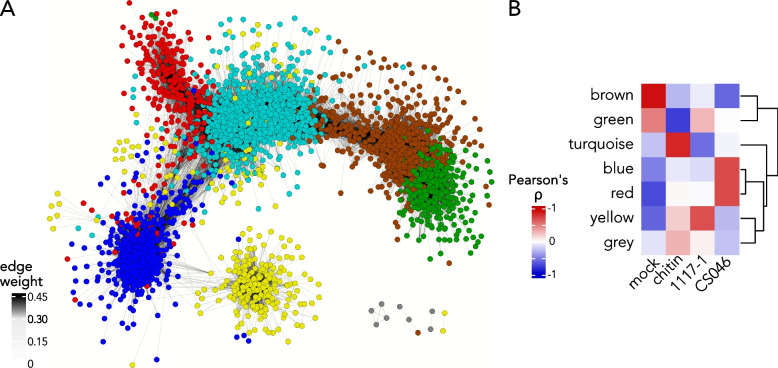
WGCNA yields a discrete co-expression module associated with EBDC defense. **A** Visualization of the weighted gene co-expression network analysis (WGCNA). Nodes represent genes colored according to co-expression module membership. Edges indicate a measurable co-expression relationship with weaker connections having increasingly transparent lines. **B** Heatmap showing module-trait relationships. Rows represent co-expression modules, and columns represent the experimental treatment. Heatmap cells are colored according to the Pearson’s correlation coefficient r between a co-expression module eigengene and an experimental treatment class. Row dendrogram illustrates hierarchical clustering results for similar co-expression modules

To identify key regulatory genes of EBDC resistance in tomato, we investigated whether the distinct transcriptional modules can be associated with specific treatments, with particular focus on a transcriptional module of defense-related genes associated with CS046 treatment. To filter out genes generally not involved in the defense responses and gain more insight into the EBDC defense response, we performed a weighted gene correlation network analysis to identify the co-expression networks underlying the CS046 defense response. 4644 genes passed our thresholds for mean expression and variance (Table S3). After filtering for edge weight, we yielded a gene co-expression network with 3430 genes placed in 8 co-expression modules, and over 200,000 co-expression relationships. The co-expression modules have distinct GO term enrichment, indicating discrete biological activities associated with specific co-expression modules (Table S1). Upon visualization the co-expression network, the boundaries between the co-expression modules are well-defined (Fig. 3a). There are 5 significantly correlated co-expression modules with Pearson’s correlations (*r*) higher than 0.65 (*p* < 0.01) associated with each experimental treatment class; the brown module (*r* = 0.93) for the mock treatment, the turquoise module (*r* = 0.84) for chitin treatment, the red (*r* = 0.70) and blue (*r* = 0.70) modules for the CS046 treatment, and the yellow module (*r* = 0.67) for the 1117–1 treatment (Fig. 3b). Among the 2 co-expression modules that are significantly correlated to the CS046 treatment, the red module can be assigned to photosynthesis-related activities, the blue module with defense responses (Table S1). To further confirm the assignment of the blue module to the defense response, we performed a Fisher’s exact test to evaluate the enrichment of genes within the co-expression modules for genes regulated by MYC2, a master regulator of necrotrophic defense responses in tomato [65]. Genes from the blue module have 67% higher odds of being MYC2-regulated (list from Du et al*.* 2017), and is the only co-expression module with statistically significant enrichment (FDR < 0.05). The blue module also has the highest average edge weight for any module in the network, with 0.24, 0.17, and 0.18 for the blue, yellow, and turquoise modules respectively.

### Nearly five-fold more predicted hub genes in the CS046-associated co-expression network module than the 1117–1-associated network

To identify the genes with the strongest influence on the topology of the defense-related co-expression network, we calculated network eigenvector centrality for each gene in a given co-expression module [66]. We found 20, 53, and 94 genes with high eigenvector centrality for the yellow, turquoise, and blue co-expression modules respectively (Table S4). For the yellow module’s defense-related hub genes, four genes are involved in lignin biosynthesis, three secondary metabolite biosynthesis genes, and one gene each of proteases, chitinases, ABC transporters, glutathione transferases, ABA-responsive genes, RLKs, and TFs. Of the turquoise hub genes that are defense-related, 20 genes are involved in cell wall remodeling, 8 molecule transport genes, 3 hormone response genes, 2 secondary metabolite biosynthesis genes, 2 RLK genes, and one gene each for calmodulin-binding-like proteins, protease inhibitors, lignin biosynthesis, stress responses, trichome development, and TFs. Of the blue module genes that are defense-related, 14 are associated with secondary metabolite biosynthesis, 12 TFs, 7 transport genes, 6 hormone response genes, 6 glutathione S-transferases, 6 stress response genes, 5 protein stress genes, 4 calmodulin-like genes, 4 lignification genes, 3 hormone biosynthesis genes, 3 signal transduction genes, 2 glycosyltransferase genes, 2 cell wall remodeling genes, and one gene each of ABC transporters, cuticle biosynthesis, MAP kinases, PCD regulators, protein synthesis, RLKs, senescence, and wounding responses. 12 of the 94 hub genes for the blue module are TFs; five *ERFs, SlERF.H14* (Solyc05g052410), *SlERF.D3* (Solyc01g108240), *SlERF.D4* (Solyc10g050970), *SlERF.D5* (Solyc04g012050), and *SlERF.D6* (Solyc04g071770); one myeloblastosis *(MYB), SlMYB79* (Solyc05g053150); one *WRKY, SlWRKY45* (Solyc08g067360); one homeodomain-leucine zipper (HD-ZIP), *SlHOX6* (Solyc03g082550); one *NAM/ATAF1/2/CUC2 (NAC), JA2* (Solyc12g013620); one DNA binding with one finger *(Dof), SlDof2.1* (Solyc06g075370); one Nuclear Transcription Factor, X-Box Binding 1 *(NFX1), NFXL1* homolog (Solyc03g118420); and one basic leucine zipper *(bZIP), SlbZIP07* (Solyc01g100460). The yellow and turquoise modules each have one TF each amongst their hub genes, *SlWRKY16* (Solyc02g032950) and *SlERF.H1* (Solyc06g065820) respectively.

### Gene regulatory network predicts significant functional specialization of the co-expression network hub gene transcription factors

**Fig. 4:**
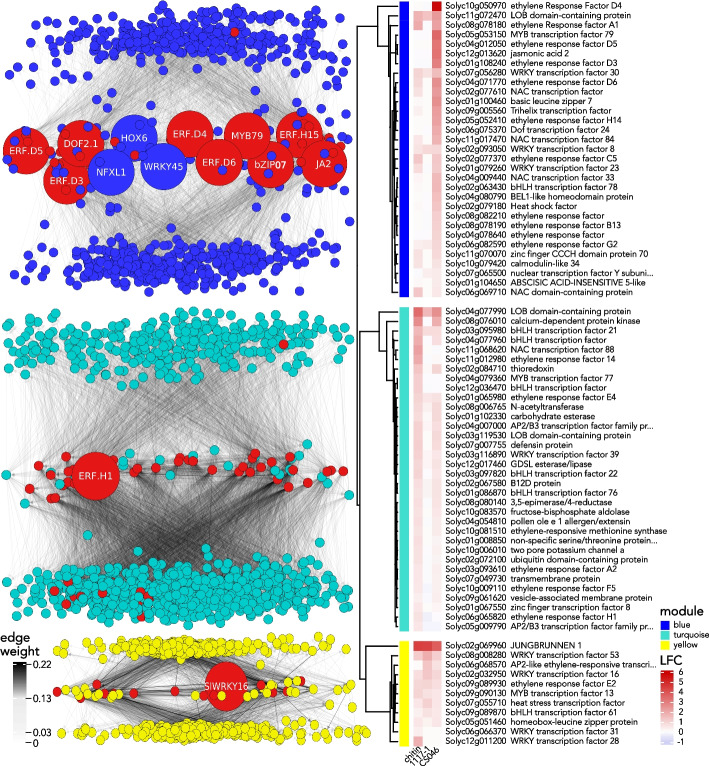
Blue module regulatory hubs show specific induction, while turquoise and yellow module regulatory hubs demonstrate generalized induction. The directional gene regulatory network (GRN) subnetworks showcase the GRN hub genes: blue for EBDC defense-associated, yellow for EBDC susceptibility-associated, and turquoise for chitin-associated co-expression modules with adjacent clustered heatmaps that are colored based on their log2 fold change (LFC). In the networks, nodes symbolize genes with their colors indicating module membership. Large nodes, annotated with their protein names, represent co-expression hub genes, while red nodes denote GRN hub genes

We constructed a directed GRN using the same input matrix previously used to generate the co-expression network to predict potential regulatory targets of the hub gene TFs (Fig. 4). The GRN subnetworks regulated by each hub gene contains hundreds of genes, and therefore we used GO term enrichment to summarize their putative biological functions (Table S1). Among the blue module GRN hub gene TFs, all have enriched gene ontologies, with only GO terms for hormone responses shared between them all, largely due to *NFXL1* having GO terms for only ET signaling and stomata movement. Our GRN predicts that the six D clade ERFs regulate ~ 47% of the genes within the blue module (Table S5). All of the predicted D clade ERF gene regulatory subnetworks have enriched gene ontologies, notably with GO terms for secondary metabolism and hormone responses being shared across all. The enriched GO terms for the *SlERF.D1* and *SlERF.D4* GRN subnetworks are similar to each other, with enriched GO terms for phenylpropanoid, coumarin, and jasmonic acid metabolism, protease and hydrolase inhibition, and responses to fungi. Still, *SlERF.D1* and *SlERF.D4* are divergent from the other D clade *ERF*s, with uniquely enriched GO terms for brassinosteroid biosynthesis and responses to vitamin B2 in *SlERF.D1,* and anthocyanin biosynthesis, stomata regulation, and xenobiotic transport for *SlERF.D4*. The four remaining D clade *ERF*s share enriched GO terms for ET signaling, stress responses, and toxin degradation, but the more functional overlap exists between *SlERF.D2, SlERF.D5,* and *SlERF.D6*, which additionally share enriched GO terms for auxin responses and abiotic stress. Excluding *NFXL1*, the remaining hub genes share enriched GO terms for toxin degradation, jasmonic acid, and phenylpropanoid biosynthesis. A particularly strong overlap exists between *JA2, SlDof2.1, SlbZIP07, and SlMYB79,* with additional shared enriched GO terms for ET and auxin signaling, coumarin biosynthesis, response to fungi, and sulfur homeostasis. *SlERF.H14* and *SlWRKY45* have additional enriched GO terms for abiotic stress in their GRN subnetworks, as well as specifically enriched GO terms for protein folding and abiotic stress for *SlERF.H14*, and stomatal movement and lignification for *SlWRKY45*. Our GRN predicts that the turquoise module’s single hub gene that is also a TF, *SlERF.H1*, regulates ~ 33% of the turquoise module (Table S5), and has enriched GO terms almost exclusively related to cell wall remodeling, as well as gibberellin responses (Table S1). The yellow module’s single co-expression hub gene that is also a TF, *SlWRKY16*, is predicted in our GRN to regulate ~ 23% of the yellow module (Table S5), and while its regulatory subnetwork has no significantly enriched GO terms, the most highly enriched GO terms are for biotic stress, defense responses, and toxin responses.

### Network statistics predict the global regulators of the defense-associated gene regulatory network

To identify global regulators of the GRN, we filtered the global GRN for co-expression module membership, and calculated network eigenvector centrality for each gene in a given GRN subnetwork. While these genes may not have a direct role in the biological activity of the co-expression module, they are predicted to regulate the regulators due to their basal role in the global regulatory hierarchy [66]. We calculated 40 hub genes within the blue module GRN (Table S4). The gene with highest eigenvector centrality in the blue module GRN is zinc finger CCCH domain-containing protein 70 (*SlC3H70*, Solyc11g070070), a gene family known to be responsive to biotic and abiotic stresses [67]. 9 genes are predicted to be hub genes in both the co-expression and gene regulatory networks, *SlbZIP07, SlERF.D3-6, SlERF.H14, SlMYB79, SlDof2.1,* and *JA2* (Fig. 4). 55 genes were predicted to be hub genes for the turquoise module GRN (Table S4). *SlERF.H1* is the only gene predicted to be hub genes for both the co-expression and gene regulatory networks, implying a crucial regulatory role (Fig. 4). The gene with the highest eigenvector centrality in the turquoise module GRN is basic helix-loop-helix 22 (*SlbHLH02*2, Solyc03g097820). 14 genes were predicted to be hub genes for the yellow module GRN (Table S4). The only gene predicted to be hub genes in both the co-expression and gene regulatory networks is *SlWRKY16* (Fig. 4). It is also the gene with the highest eigenvector centrality in the yellow module GRN and is predicted to regulate every other co-expression network hub gene in the yellow module.

## Discussion

### Weak immune induction during compatible pathogenesis at 3 hpi

We investigated the expression of genes associated with canonical PTI and ETI signaling pathways. Gene induction in almost all immune pathways is observed in the CS046 treatment, but immune induction in the 1117–1 treatment is notably weaker, suggesting compromised host defense signaling (Fig. 2). Pathogenic effectors are often the means by which host defenses can be suppressed [68], and EBDC genomes are known to encode nearly 200 candidate effector proteins, 4 of which are experimentally validated to contribute to virulence [69–71]. In necrotrophs, NLR-mediated detection of effectors might overstimulate immunity and promote susceptibility [72], with the notable exception of the *Alternaria* apple scab pathosystem in which recognition of a necrotrophic effector by an NLR induces effective immunity [73]. It remains inconclusive whether 1117–1 secretes an effector that overstimulates plant immunity as an infection strategy due to the overall lack of immune induction in this treatment. Our data suggests that neither resistance nor susceptibility to EBDC involves expression of NLR genes at 3 hpi. If 1117–1 suppresses plant immunity with a canonical pathogenic effector, CS046 either does not possess it, or an NLR is not required as an effective countermeasure against it. Hormone-responsive genes, calcium signaling, and TFs showed the greatest amplitude of induction and diversity in response among the defense gene categories we investigated (Fig. 2). Specifically, *ERFs* seem to have consistently high induction in CS046 and chitin treated tomatoes. *SlERF.C1* (Solyc05g051200) is the only *ERF* that is a DEG in the 1117–1 treatment (Table S2). It has nearly equal log_2_ fold change for all experimental treatments, and therefore has little explanatory power for the weak defense response of the 1117–1 treatment. The strong induction of *ERFs* in the CS046 treatment as a host defense strategy is consistent with our understanding of necrotrophic defense signaling [65], including specific expression of a whole *ERF* clade [74], but specific induction the D clade ERFs is unreported in other pathosystems.

### Network analyses detect response modules to EBDC and chitin

We generated a co-expression network to investigate the topology of the transcriptional landscape during EBDC defense. We yielded one co-expression module associated with the 1117–1 and chitin treatments, the yellow and turquoise modules respectively, and two modules associated with the CS046 treatment, the red and blue modules (Fig. 3). To allow better interpretation of the module’s functions we expanded the functional annotations of *S. lycopersicum* and specifically curated sets of genes likely to be defense associated (Table S1, Data S1). The co-expression modules can be assigned to distinct biological activities (Table S1). The blue module is primarily associated with secondary metabolite biosynthesis and hormone signaling, whereas the red module contains photosynthesis-related genes, and therefore we infer that the blue module represents the core EBDC defense response. The turquoise module is enriched for GO terms for cell wall remodeling, GA signaling, and mitosis. This is consistent with the effect of chitin treatment on rice, which was found to mediate resistance to a fungal pathogen by inducing compositional changes in the cell wall [75]. The yellow module is largely comprised of generic stress responses, but without ET/JA signaling, and lesser induction of defense-related genes compared to the blue module. Assignment of the blue, turquoise, and yellow modules to defense-associated co-expression subnetworks is consistent with previous findings in *A. thaliana* that found core fungal necrotroph response genes to be RLKs, cell wall remodeling, *WRKY*, *NAC*, and *MYB* TFs, glycosyltransferases, calcium-signaling proteins, hormone response genes, glutathione transferases, nutrient scavenging, HR suppression, ABC1 proteins, lipid transfer proteins, heat shock factors, and thioredoxins [76], all of which are major components of these three co-expression modules.

### EBDC-associated co-expression network hub genes are both known and novel in plant-pathogen interactions

High eigenvector centrality is a favored network statistic to predict transcription network hubs due to its detection of biologically meaningful genes that other centrality statistics fail to detect [77]. The EBDC-associated co-expression modules yield several hub genes with previously described direct roles in plant immunity, and genes with no confirmed defense role (Table S4). In the blue module, stress-associated protein (SlSAP12, Solyc02g087210) is an AN-1 zinc finger protein that negatively regulates nematode parasitism [78], AOC (Solyc02g085730) plays a crucial step in the JA biosynthesis pathway [79], PORK1 (Solyc03g123860) is an RLK crucial for systemin-mediated defense to *B. cinerea* [80], heat shock protein 70 7 (SlHSP70-7, Solyc03g117630) is a protein family that stabilizes defense-related proteins [81], and phytosulfokine 3-like (PSK3L, Solyc02g092120), is a pentapeptide damage-associated molecular pattern that positively regulates defense to *B. cinerea* infection by inducing a Ca^2+^ burst upon perception [82]. In the yellow module, chitinases (Solyc04g072000) are well-known defense-related proteins [83], and an Omega-6 fatty acid desaturase (Solyc04g040130) had high expression in another comparative transcriptomics study in the compatible pathosystem [84]. In the turquoise module, *SlERF.H1* was previously implicated in defense to the fungal pathogen *Rhizopus nigricans* through the expression of *PR5*, phenylalanine ammonia-lyase (PAL), and chitinase genes [85]. The turquoise module contains 2 of the 5 chitinase genes in the co-expression network, and 2 of the 3 PAL genes (Table S3). PAL genes catalyze the biosynthesis of cinnamic acid and are involved in at least three different defense-related biosynthesis pathways: suberin, phenylpropanoids, and SA biosynthesis [86]. Cinnamic acid and its hydroxylated derivatives also display direct antifungal activity, and foliar application 24 hpi reduces the severity of EBDC symptoms in tomato [87]. The genes associated with biosynthesis of cinnamic acid and its derivatives are not DEGs in any treatments, but downstream reactions that consume them are DEGs in all treatments. This suggests that at 3 hpi, these biosynthesis pathways are terminating at suberin, cuticle, and lignin biosynthesis, and accumulation of cinnamic acid derivatives to mediate EBDC defense is not inferred from our analysis.

Crucially, we observe evidence that due to SA primacy during SA/JA antagonism, JA signaling may be attenuated during 1117–1 infection compared with CS046 infection since a homolog of an SA biosynthesis gene, *AIM1* (Solyc08g068390), is upregulated during 1117–1 infection and is in the yellow module (Table S3) [88]. In contrast, a UDP-xylose phenolic glycosyltransferase (Solyc08g006330) that negatively regulates methyl salicylate biosynthesis is significantly upregulated during CS046 treatment, and is in the blue module (Table S3) [89].

### Gene regulatory network predicts stronger complexity of defense network regulation compared to the chitin- or the susceptibility-associated networks

The turquoise and yellow modules both have just one TF that is predicted to be a hub gene for both the co-expression and gene regulatory networks, whereas the blue module has nine, indicating a more complex regulatory landscape associated with EBDC defense than for bare elicitation or EBDC susceptibility (Table S4). The gene with the highest eigenvector centrality in the blue module GRN is *SlC3H70.* Though it is not a hub gene in the co-expression network, the zinc finger CCCH domain-containing gene family are known to be responsive to biotic and abiotic stresses [67]. Many blue module GRN hub genes have well-known roles in plant immunity. The blue module GRN hub genes with the second- and third-highest eigenvector centrality are *SlERF.A1* (Solyc08g078180) and *JA2-like* (Solyc07g063410), both of which directly contribute to defense to *B. cinerea* in tomatoes [65, 90], and other defense-related *ERFs,* for example, *SlERF.C6* (Solyc02g077370), implicated in abiotic stress in previous studies [91]. 9 genes are predicted to be hub genes in both the co-expression and gene regulatory networks (Fig. 4), notably *SlERF.D3-6, SlMYB79* which is strongly induced during *Passalora fulva* defense in *Cf-12* tomatoes [92], *JA2,* known to participate in stomatal closure during pathogen defense [93], and *SlWRKY45,* a gene known to bind and inhibit the promoter of allene oxide cyclase (AOC) to repress JA biosynthesis in the root-knot nematode pathosystem [94], but induces JA biosynthesis in the presence of ergosterol/squalene [95]. The defense roles of the D clade *ERF*s – *SlERF.D3-6* – are relatively uncharacterized. It has been shown that *SlERF.D4-6* are upregulated during ETI of *Pseudomonas syringae* pv. *tomato* (*Pst*) strain DC3000 infection [96], and *SlERF.D2, SlERF.D6,* and *SlERF.D7* expression is induced in response to *B. cinerea* infection of red ripe tomato fruits but not wounding [97], suggesting a role in the defense response specific to pathogen detection. None of these TFs have been implicated in EBDC defense before, and we report a role in biotic stress in tomato for *SlbZIP07, NFXL1, SlERF.D3* and *SlERF.H14* for the first time. The D clade *ERF*s are 5 of the 6 most strongly induced hormone response genes in the CS046 treatment (Fig. 2), they are specifically induced as a transcriptional module in CS046 treatment (Table S2), and have novel implication in EBDC defense. The overlap of the GRN and co-expression network results indicate a major role for ET-regulation, and regulatory roles for several more genes. The most highly upregulated turquoise module GRN hub gene *ERF* is *SlERF014* (Solyc11g012980), a gene that occupies the H clade of ERFs in the Pirrello et al*.* 2012 nomenclature scheme [64, 98], is a known susceptibility factor to *Botrytis cinerea* [99], and is notably uninduced in EBDC-treated samples. Notable turquoise module GRN hub genes with known defense roles include three *ERF*s, *SlERF.H1* [85], *SlERF.F5* (Solyc10g009110) [100], *SlWRKY39* (Solyc03g116890) [101], defensin 9 (*SlDEF9,* Solyc07g007755) [102], and zinc-finger protein 62 (*SlZF-62,* Solyc06g075780), whose homolog in *A. thaliana* is upregulated in response to chitin [103]. The yellow module yielded one TF as a hub gene in its co-expression network, *SlWRKY16.* Though its defense role is unclear, it is upregulated during ETI of *Pst* DC3000 [96]. It is also the gene with the highest eigenvector centrality in the yellow module GRN*,* and in our dataset, its strongest regulatory target is a chitinase and it regulates many RLKs and several lignification genes (Table S5), but it is expressed evenly in all treatments, producing no explanatory power for susceptibility to 1117–1 and defense to CS046.

### Putative susceptibility factors as yellow module hub genes

Several notable susceptibility factors are either components of the susceptibility-associated yellow module. In general, the rapid PCD of HR is considered a susceptibility factor in necrotrophic pathosystems in tomato [104], as well as the slow PCD of leaf senescence, both of which are induced by successful necrotrophic pathogens due to cross-talk between these cell death pathways [105]. Enrichment analysis using Fisher’s Exact Test for the list of HR-associated genes in tomato generated by Etalo et al. show a 50.3% increase in probability for an HR-associated gene to have yellow module membership than blue module membership [106]. The yellow module also contains several genes known to promote leaf senescence, for example *SlNAP2* (Solyc04g005610) (Table S4) [107]. *SlWRKY16* and *SlWRKY53* are both yellow module GRN hub genes, and *SlWRKY16* is a co-expression network hub gene as well (Table S4). Both are orthologs of two well-studied positive regulators of senescence in *A. thaliana, AtWRKY6* and *AtWRKY53* respectively [108, 109]. *SlWRKY17*, a yellow module gene, is also an ortholog of *AtWRKY6* with exclusive induction in 1117–1-treated tomatoes (Table S3). Both *AtWRKY53* and *AtWRKY6* induce SA signaling to positively regulate leaf senescence in *A. thaliana* [108, 110]. In *A. thaliana,* SA is a known promoter of pathogen-induced HR [111], and leaf senescence [110]. Although comparatively under-studied in tomato, SA in has been linked to HR [106], but as noted by Guo et al. 2021, SA-induced leaf senescence is woefully under-studied in plant systems outside *A. thaliana* [112]. SA has nonetheless been established as a negative regulator of necrotroph defense. Knockout lines of the master regulator of SA-signaling, *SlNPR1*, enhances defense to the necrotrophic fungus *B. cinerea* [113]. Enrichment analysis shows that the list of SA-associated susceptibility genes from Li et al*.* 2021 are 47% more likely to have yellow module membership than blue module membership. JA exhibits a crucial role in preventing SA-induced PCD programs due to SA/JA antagonism. In *A. thaliana,* JA antagonism of SA has been demonstrated to negatively regulate leaf senescence [109], and HR [114]. JA exerts antagonistic primacy against SA in the presence of ET [17], and may counteract the effects of HR- and senescence-promoting gene expression. For example *SlWRKY16* and *SlWRKY53* are significantly upregulated in all treatments, as well as *JUNGBRUNNEN1 (JUB1*, Solyc02g069960) (Table S3), a gene known to promote leaf senescence in tomato [115], but several lines of evidence suggest that the CS046 treatment accumulates high levels of JA to counteract the senescence-promotion. For example, genes that are induced by both JA and *SlMYC2*, the master regulator of JA signaling in tomato, are significantly enriched in the blue module only, and significantly depleted in the yellow module, with a 579% increase in probability for an *SlMYC2*-induced gene to have blue module membership than yellow module membership [65]. Furthermore, the most upregulated gene in the CS046 treatment is *JASMONATE-INDUCED OXYGENASE 3* (*JOX3,* Solyc10g076670), a gene that catabolizes excessive JA and indicates high levels of JA [116], but has little expression in all other treatments (Table S3). It is tempting to speculate that this is a major driver of 1117–1 virulence; chitin is sufficient to induce promoters of leaf senescence, and failure to induce JA/ET pathways in the presence of chitin and other PAMPs may be sufficient to derepress leaf senescence, and therefore promote pathogen virulence. Previous studies have shown that an *A. solani* isolate secretes proteinaceous effectors that increase virulence in tomato, and induce expression of senescence genes in *Nicotiana benthamiana* 4 days post infection, specifically *SEN4, SAG12,* and *DHAR1* [70, 71].

### The specificity of responses implicates calmodulins and ethylene response factors as mediators of the rapid inferred JA accumulation

Plants rapidly biosynthesize JA and ET in response to stress, and previous studies have identified ACC as an enriched secondary metabolite between CS046- and 1117–1-treated Heinz 1706 tomatoes, highlighting a major role for ET in this pathosystem [29]. The upstream position of ERFs in the signal cascade makes them compelling subjects of analysis [117]. Tomato responds to herbivory with a rapid Ca^2+^ burst that activates SlCaM2 (Solyc10g081170) to bind SlERF16 (Solyc12g009240), afterwards inducing expression of itself and JA biosynthesis genes, increasing JA and ET by 5- and tenfold respectively within 15 min [118]. Downstream JA signaling events are largely controlled by the master regulator of JA responses MYC2 (Solyc08g076930) [119], whose target genes are constitutively repressed by JAZ proteins, and derepressed in the presence of JA [120, 121]. In our dataset, we find *SlCaM2/3/5* (Solyc10g081170, Solyc10g077010, Solyc12g099990) in the turquoise module with relatively high gene expression (FDR < 0.05, LFC < 1) in both the chitin and CS046 treatments (Table S2). We do not, however, observe differential expression of *SlERF16*, which should be induced by itself and JA in a feedback loop [118, 122]. This could be due to 3 hpi being an inappropriate time point to measure *SlERF16* expression. Still, since *SlERF16* was studied in herbivory studies, this result may highlight a longstanding gap in our knowledge of JA/ET signaling in defense responses – the eminent specificity of the responses [65]. Additionally, SlERF.D3-6 all have predicted calmodulin binding domains. Caution should be taken against strong conclusions of the defense role of D clade ERFs at this early stage, however. Negative immune regulators are also induced by JA, for example JAZ and JOX3, and a negative immune regulation role is not ruled out by our analyses. Downstream studies that knockout and over-express the D clade ERFs, and other defense hub genes predicted in this study, will clarify their role during EBDC pathogenesis.

### Supplementary Information

Supplementary Material 1.

Supplementary Material 2.

Supplementary Material 3.

Supplementary Material 4.

Supplementary Material 5.

Supplementary Material 6.

Supplementary Material 7.

## Data Availability

The RNA-Seq datasets generated and analysed during the current study are available in the NCBI Sequence Read Archive (SRA) repository, accession PRJEB68314. All other data generated during this study are included in this published article and its supplementary information files. All scripts used for the analyses are available at Zenodo 10.5281/zenodo.10642413

## References

[1] Lukyanenko AN, Kalloo G (1991). Disease Resistance in Tomato. GeneticImprovement of Tomato.

[2] Zalom FG. Pests, endangered pesticides and processing tomatoes. Acta Hortic. 2003;613:223–33.

[3] Foolad MR, Merk HL, Ashrafi H (2008). Genetics, genomics and breeding of late blight and early blight resistance in tomato. Crit Rev Plant Sci.

[4] Woudenberg JHC, Truter M, Groenewald JZ, Crous PW (2014). Large-spored Alternaria pathogens in section Porri disentangled. Stud Mycol.

[5] Woudenberg JHC, Seidl MF, Groenewald JZ, de Vries M, Stielow JB, Thomma BPHJ (2015). Alternaria section Alternaria: Species, formae speciales or pathotypes?. Stud Mycol.

[6] Schmey T, Tominello-Ramirez CS, Brune C, Stam R (2024). Alternaria diseases on potato and tomato. Mol Plant Pathol.

[7] Vandecasteele M, Landschoot S, Carrette J, Verwaeren J, Höfte M, Audenaert K (2018). Species prevalence and disease progression studies demonstrate a seasonal shift in the Alternaria population composition on potato. Plant Pathol.

[8] Adhikari P, Oh Y, Panthee DR (2017). Current status of early blight resistance in tomato: an update. Int J Mol Sci.

[9] Fungicide Resistance Action Committee. Pathogen Risk List. 2019. https://www.frac.info/docs/default-source/publications/pathogen-risk/frac-pathogen-list-2019.pdf?sfvrsn=763d489a_2. Accessed 3 Aug 2023.

[10] Tan K-C, Oliver RP, Solomon PS, Moffat CS (2010). Proteinaceous necrotrophic effectors in fungal virulence. Funct Plant Biol.

[11] Glazebrook J (2005). Contrasting mechanisms of defense against biotrophic and necrotrophic pathogens. Annu Rev Phytopathol.

[12] Tsuda K, Katagiri F (2010). Comparing signaling mechanisms engaged in pattern-triggered and effector-triggered immunity. Curr Opin Plant Biol.

[13] Martin RL, Boulch PL, Clin P, Schwarzenberg A, Yvin J-C, Andrivon D (2020). A comparison of PTI defense profiles induced in Solanum tuberosum by PAMP and non-PAMP elicitors shows distinct, elicitor-specific responses. PLoS One.

[14] Yoo H, Greene GH, Yuan M, Xu G, Burton D, Liu L (2020). Translational regulation of metabolic dynamics during effector-triggered immunity. Mol Plant.

[15] Caarls L, Pieterse CMJ, Van Wees SCM (2015). How salicylic acid takes transcriptional control over jasmonic acid signaling. Front Plant Sci.

[16] Robert-Seilaniantz A, Grant M, Jones JDG (2011). Hormone crosstalk in plant disease and defense: more than just jasmonate-salicylate antagonism. Annu Rev Phytopathol.

[17] Leon-Reyes A, Du Y, Koornneef A, Proietti S, Körbes AP, Memelink J (2010). Ethylene signaling renders the jasmonate response of arabidopsis insensitive to future suppression by salicylic acid. Mol Plant Microbe Interact.

[18] Kamakshi K, MohanaPrasad J, Muthamilarasan M, Radhakrishnan N (2023). Foliar application of Methyl Jasmonate Methyl Ester elicits differential antioxidant defence and expression of defence-related genes against early blight disease of tomato. J Phytopathol.

[19] Spletzer ME, Enyedi AJ (1999). Salicylic acid induces resistance to alternaria solani in hydroponically grown tomato. Phytopathology.

[20] Ali S, Ganai BA, Kamili AN, Bhat AA, Mir ZA, Bhat JA (2018). Pathogenesis-related proteins and peptides as promising tools for engineering plants with multiple stress tolerance. Microbiol Res.

[21] Lawrence CB, Joosten MHAJ, Tuzun S (1996). Differential induction of pathogenesis-related proteins in tomato byAlternaria solaniand the association of a basic chitinase isozyme with resistance. Physiol Mol Plant Pathol.

[22] Lawrence CB, Singh NP, Qiu J, Gardner RG, Tuzun S (2000). Constitutive hydrolytic enzymes are associated with polygenic resistance of tomato to Alternaria solani and may function as an elicitor release mechanism. Physiol Mol Plant Pathol.

[23] Dita MA, Brommonschenkel SH, Matsuoka K, Mizubuti ESG (2007). Histopathological study of the alternaria solani infection process in potato cultivars with different levels of early blight resistance. J Phytopathol.

[24] Vleeshouwers VGAA, van Dooijeweert W, Govers F, Kamoun S, Colon LT (2000). The hypersensitive response is associated with host and nonhost resistance to Phytophthora infestans. Planta.

[25] Brouwer SM, Brus-Szkalej M, Saripella GV, Liang D, Liljeroth E, Grenville-Briggs LJ (2021). Transcriptome analysis of potato infected with the necrotrophic pathogen Alternaria solani. Plants.

[26] Zheng L, Yang P, Niu Z, Tian M, Wang J, Sun C (2022). Dissecting in vivo responses of phytohormones to Alternaria solani infection reveals orchestration of JA- and ABA-mediated antifungal defenses in potato. Hortic Res..

[27] Sajeevan RS, Abdelmeguid I, Saripella GV, Lenman M, Alexandersson E (2023). Comprehensive transcriptome analysis of different potato cultivars provides insight into early blight disease caused by Alternaria solani. BMC Plant Biol.

[28] Schmey T, Small C, Einspanier S, Hoyoz LM, Ali T, Gamboa S, et al. Small-spored Alternaria spp. (section Alternaria) are common pathogens on wild tomato species. Environ Microbiol. 2023. 10.1111/1462-2920.16394. 10.1111/1462-2920.16394 37171093

[29] Muñoz Hoyos L, Anisha WP, Meng C, Kleigrewe K, Dawid C, Hückelhoven R, et al. Untargeted metabolomics reveals PTI-associated metabolites. Plant Cell Environ. 2023. 10.1111/pce.14794. 10.1111/pce.14794 38164085

[30] Bernasconi A, Lorrain C, Flury P, Alassimone J, McDonald BA, Sánchez-Vallet A (2023). Virulent strains of Zymoseptoria tritici suppress the host immune response and facilitate the success of avirulent strains in mixed infections. PLOS Pathog.

[31] Kahlon PS, Förner A, Muser M, Oubounyt M, Gigl M, Hammerl R, et al. Laminarin-triggered defence responses are geographically dependent in natural populations of Solanum chilense. J Exp Bot. 2023;74(10):erad087. 10.1093/jxb/erad087 PMC10199122 36880316

[32] Nirenberg H (1976). Untersuchungen über die morphologische und biologische Differenzierung in der Fusarium-Sektion Liseola.

[33] Bolger AM, Lohse M, Usadel B (2014). Trimmomatic: a flexible trimmer for Illumina sequence data. Bioinformatics.

[34] Hosmani PS, Flores-Gonzalez M, van de Geest H, Maumus F, Bakker LV, Schijlen E (2019). An improved de novo assembly and annotation of the tomato reference genome using single-molecule sequencing, Hi-C proximity ligation and optical maps.

[35] Kim D, Paggi JM, Park C, Bennett C, Salzberg SL (2019). Graph-based genome alignment and genotyping with HISAT2 and HISAT-genotype. Nat Biotechnol.

[36] Liao Y, Smyth GK, Shi W (2014). featureCounts: an efficient general purpose program for assigning sequence reads to genomic features. Bioinformatics.

[37] Love MI, Huber W, Anders S (2014). Moderated estimation of fold change and dispersion for RNA-seq data with DESeq2. Genome Biol.

[38] Zhu A, Ibrahim JG, Love MI (2019). Heavy-tailed prior distributions for sequence count data: removing the noise and preserving large differences. Bioinformatics.

[39] Benjamini Y, Hochberg Y (1995). Controlling the false discovery rate: a practical and powerful approach to multiple testing.

[40] Gu Z (2022). Complex heatmap visualization. iMeta.

[41] Shannon P, Markiel A, Ozier O, Baliga NS, Wang JT, Ramage D (2003). Cytoscape: a software environment for integrated models of biomolecular interaction networks. Genome Res.

[42] Maere S, Heymans K, Kuiper M (2005). BiNGO: a Cytoscape plugin to assess overrepresentation of Gene Ontology categories in Biological Networks. Bioinformatics.

[43] Törönen P, Holm L (2022). PANNZER—A practical tool for protein function prediction. Protein Sci.

[44] Liu Y, Zhang Y, Liu X, Shen Y, Tian D, Yang X (2023). SoyOmics: A deeply integrated database on soybean multi-omics. Mol Plant.

[45] Liu Z, Ren Z, Yan L, Li F (2022). DeepLRR: an online webserver for leucine-rich-repeat containing protein characterization based on deep learning. Plants.

[46] Sakamoto T, Deguchi M, Brustolini OJ, Santos AA, Silva FF, Fontes EP (2012). The tomato RLK superfamily: phylogeny and functional predictions about the role of the LRRII-RLK subfamily in antiviral defense. BMC Plant Biol.

[47] Thumuluri V, Almagro Armenteros JJ, Johansen AR, Nielsen H, Winther O (2022). DeepLoc 2.0: multi-label subcellular localization prediction using protein language models. Nucleic Acids Res.

[48] Hallgren J, Tsirigos KD, Pedersen MD, Armenteros JJA, Marcatili P, Nielsen H, et al. DeepTMHMM predicts alpha and beta transmembrane proteins using deep neural networks. 2022:2022.04.08.487609. 10.1101/2022.04.08.487609.

[49] Eddy SR (2011). Accelerated Profile HMM Searches. PLOS Comput Biol.

[50] Lu S, Wang J, Chitsaz F, Derbyshire MK, Geer RC, Gonzales NR (2020). CDD/SPARCLE: the conserved domain database in 2020. Nucleic Acids Res.

[51] Kang W-H, Yeom S-I (2018). Genome-wide identification, classification, and expression analysis of the receptor-like protein family in tomato. Plant Pathol J.

[52] Gíslason MH, Nielsen H, Almagro Armenteros JJ, Johansen AR (2021). Prediction of GPI-anchored proteins with pointer neural networks. Curr Res Biotechnol.

[53] Wu J, Wang J, Pan C, Guan X, Wang Y, Liu S (2014). Genome-wide identification of MAPKK and MAPKKK gene families in tomato and transcriptional profiling analysis during development and stress response. PLoS ONE.

[54] Tian F, Yang D-C, Meng Y-Q, Jin J, Gao G (2020). PlantRegMap: charting functional regulatory maps in plants. Nucleic Acids Res.

[55] Liu M, Pirrello J, Chervin C, Roustan J-P, Bouzayen M (2015). Ethylene control of fruit ripening: revisiting the complex network of transcriptional regulation. Plant Physiol.

[56] Emms DM, Kelly S (2019). OrthoFinder: phylogenetic orthology inference for comparative genomics. Genome Biol.

[57] Camacho C, Coulouris G, Avagyan V, Ma N, Papadopoulos J, Bealer K (2009). BLAST+: architecture and applications. BMC Bioinformatics.

[58] Papadopoulos JS, Agarwala R (2007). COBALT: constraint-based alignment tool for multiple protein sequences. Bioinformatics.

[59] Wickham H (2009). ggplot2: elegant graphics for data analysis.

[60] Langfelder P, Horvath S (2008). WGCNA: an R package for weighted correlation network analysis. BMC Bioinformatics.

[61] Csárdi G, Nepusz T. The igraph software package for complex network research. Complex Syst. 2006;1695(5):1695. https://www.bibsonomy.org/bibtex/bb49a4a77b42229a427fec316e9fe515.

[62] Huynh-Thu VA, Irrthum A, Wehenkel L, Geurts P (2010). Inferring regulatory networks from expression data using tree-based methods. PLoS ONE.

[63] Zhou J-M, Zhang Y (2020). Plant immunity: danger perception and signaling. Cell.

[64] Liu M, Gomes BL, Mila I, Purgatto E, Peres LEP, Frasse P (2016). Comprehensive profiling of ethylene response factor expression identifies ripening-associated ERF genes and their link to key regulators of fruit ripening in tomato. Plant Physiol.

[65] Du M, Zhao J, Tzeng DTW, Liu Y, Deng L, Yang T (2017). MYC2 orchestrates a hierarchical transcriptional cascade that regulates jasmonate-mediated plant immunity in tomato. Plant Cell.

[66] Escorcia-Rodríguez JM, Gaytan-Nuñez E, Hernandez-Benitez EM, Zorro-Aranda A, Tello-Palencia MA, Freyre-González JA (2023). Improving gene regulatory network inference and assessment: The importance of using network structure. Front Genet.

[67] Xu R (2014). Genome-wide analysis and identification of stress-responsive genes of the CCCH zinc finger family in Solanum lycopersicum. Mol Genet Genomics.

[68] Jones JDG, Dangl JL (2006). The plant immune system. Nature.

[69] Wang J, Xiao S, Zheng L, Pan Y, Zhao D, Zhang D (2022). Multiomic approaches reveal novel lineage-specific effectors in the potato and tomato early blight pathogen Alternaria solani. Phytopathol Res.

[70] Wang C, Zhang D, Cheng J, Zhao D, Pan Y, Li Q (2022). Identification of effector CEP112 that promotes the infection of necrotrophic Alternaria solani. BMC Plant Biol.

[71] Wang C, Wang J, Zhang D, Cheng J, Zhu J, Yang Z (2023). Identification and functional analysis of protein secreted by Alternaria solani. PLoS ONE.

[72] Barbacci A, Navaud O, Mbengue M, Barascud M, Godiard L, Khafif M (2020). Rapid identification of an Arabidopsis NLR gene as a candidate conferring susceptibility to Sclerotinia sclerotiorum using time-resolved automated phenotyping. Plant J.

[73] Meng D, Li C, Park H-J, González J, Wang J, Dandekar AM (2018). Sorbitol modulates resistance to alternaria alternata by regulating the expression of an NLR resistance gene in apple. Plant Cell.

[74] Thagun C, Imanishi S, Kudo T, Nakabayashi R, Ohyama K, Mori T (2016). Jasmonate-responsive ERF transcription factors regulate steroidal glycoalkaloid biosynthesis in tomato. Plant Cell Physiol.

[75] Takagi M, Hotamori K, Naito K, Matsukawa S, Egusa M, Nishizawa Y (2022). Chitin-induced systemic disease resistance in rice requires both OsCERK1 and OsCEBiP and is mediated via perturbation of cell-wall biogenesis in leaves. Front Plant Sci.

[76] Amrine KCH, Blanco-Ulate B, Cantu D (2015). Discovery of core biotic stress responsive genes in arabidopsis by weighted gene co-expression network analysis. PLoS ONE.

[77] Peng Q, Schork N (2014). Utility of network integrity methods in therapeutic target identification. Front Genet..

[78] Zhao J, Mejias J, Quentin M, Chen Y, de Almeida-Engler J, Mao Z (2020). The root-knot nematode effector MiPDI1 targets a stress-associated protein (SAP) to establish disease in Solanaceae and Arabidopsis. New Phytol.

[79] Sun J-Q, Jiang H-L, Li C-Y (2011). Systemin/jasmonate-mediated systemic defense signaling in tomato. Mol Plant.

[80] Xu S, Liao C-J, Jaiswal N, Lee S, Yun D-J, Lee SY (2018). Tomato PEPR1 ORTHOLOG RECEPTOR-LIKE KINASE1 regulates responses to systemin, necrotrophic fungi, and insect herbivory. Plant Cell.

[81] Berka M, Kopecká R, Berková V, Brzobohatý B, Černý M (2022). Regulation of heat shock proteins 70 and their role in plant immunity. J Exp Bot.

[82] Zhang H, Hu Z, Lei C, Zheng C, Wang J, Shao S (2018). A plant phytosulfokine peptide initiates auxin-dependent immunity through cytosolic Ca2+ signaling in tomato. Plant Cell.

[83] Vaghela B, Vashi R, Rajput K, Joshi R (2022). Plant chitinases and their role in plant defense: a comprehensive review. Enzyme Microb Technol.

[84] Manzo D, Ferriello F, Puopolo G, Zoina A, D’Esposito D, Tardella L (2016). Fusarium oxysporum f.sp. radicis-lycopersici induces distinct transcriptome reprogramming in resistant and susceptible isogenic tomato lines. BMC Plant Biol..

[85] Pan X-Q, Fu D-Q, Zhu B-Z, Lu C-W, Luo Y-B (2013). Overexpression of the ethylene response factor SlERF1 gene enhances resistance of tomato fruit to Rhizopus nigricans. Postharvest Biol Technol.

[86] Vogt T (2010). Phenylpropanoid Biosynthesis. Mol Plant.

[87] Nehela Y, Mazrou YSA, Taha NA, Elzaawely AA, Xuan TD, Makhlouf AH (2023). Hydroxylated Cinnamates enhance tomato resilience to alternaria alternata, the causal agent of early blight disease, and stimulate growth and yield traits. Plants.

[88] Yokotani N, Hasegawa Y, Sato M, Hirakawa H, Kouzai Y, Nishizawa Y (2021). Transcriptome analysis of Clavibacter michiganensis subsp. michiganensis-infected tomatoes: a role of salicylic acid in the host response. BMC Plant Biol..

[89] Bineau E, Rambla JL, Duboscq R, Corre M-N, Bitton F, Lugan R (2022). Inheritance of secondary metabolites and gene expression related to tomato fruit quality. Int J Mol Sci.

[90] Ouyang Z, Liu S, Huang L, Hong Y, Li X, Huang L (2016). Tomato SlERF.A1, SlERF.B4, SlERF.C3 and SlERF.A3, members of B3 group of ERF family, are required for resistance to botrytis cinerea. Front Plant Sci.

[91] Waseem M, Rong X, Li Z (2019). Dissecting the role of a basic helix-loop-helix transcription factor, SlbHLH22, under salt and drought stresses in transgenic Solanum lycopersicum L. Front Plant Sci.

[92] Xue D-Q, Chen X-L, Zhang H, Chai X-F, Jiang J-B, Xu X-Y (2017). Transcriptome analysis of the Cf-12-mediated resistance response to Cladosporium fulvum in Tomato. Front Plant Sci.

[93] Du M, Zhai Q, Deng L, Li S, Li H, Yan L (2014). Closely related NAC transcription factors of tomato differentially regulate stomatal closure and reopening during pathogen attack. Plant Cell.

[94] Huang H, Zhao W, Qiao H, Li C, Sun L, Yang R (2022). SlWRKY45 interacts with jasmonate-ZIM domain proteins to negatively regulate defense against the root-knot nematode Meloidogyne incognita in tomato. Hortic Res..

[95] Lindo L, Cardoza RE, Lorenzana A, Casquero PA, Gutiérrez S (2020). Identification of plant genes putatively involved in the perception of fungal ergosterol-squalene. J Integr Plant Biol.

[96] Pombo MA, Zheng Y, Fernandez-Pozo N, Dunham DM, Fei Z, Martin GB (2014). Transcriptomic analysis reveals tomato genes whose expression is induced specifically during effector-triggered immunity and identifies the Epk1 protein kinase which is required for the host response to three bacterial effector proteins. Genome Biol.

[97] Li S, Wu P, Yu X, Cao J, Chen X, Gao L (2022). Contrasting roles of ethylene response factors in pathogen response and ripening in fleshy fruit. Cells.

[98] Pirrello J, Prasad BN, Zhang W, Chen K, Mila I, Zouine M (2012). Functional analysis and binding affinity of tomato ethylene response factors provide insight on the molecular bases of plant differential responses to ethylene. BMC Plant Biol.

[99] Zhang H, Hong Y, Huang L, Li D, Song F (2016). Arabidopsis AtERF014 acts as a dual regulator that differentially modulates immunity against Pseudomonas syringae pv. tomato and Botrytis cinerea. Sci Rep..

[100] Álvarez-Gómez TB, Ramírez-Trujillo JA, Ramírez-Yáñez M, Suárez-Rodríguez R (2021). Overexpression of SlERF3b and SlERF5 in transgenic tomato alters fruit size, number of seeds and promotes early flowering, tolerance to abiotic stress and resistance to Botrytis cinerea infection. Ann Appl Biol.

[101] Sun X, Gao Y, Li H, Yang S, Liu Y (2015). Over-expression of SlWRKY39 leads to enhanced resistance to multiple stress factors in tomato. J Plant Biol.

[102] Nikoloudakis N, Pappi P, Markakis EA, Charova SN, Fanourakis D, Paschalidis K (2020). Structural diversity and highly specific host-pathogen transcriptional regulation of defensin genes is revealed in tomato. Int J Mol Sci.

[103] Libault M, Wan J, Czechowski T, Udvardi M, Stacey G (2007). Identification of 118 arabidopsis transcription factor and 30 ubiquitin-ligase genes responding to chitin, a plant-defense elicitor. Mol Plant Microbe Interact.

[104] Jeblick T, Leisen T, Steidele CE, Albert I, Müller J, Kaiser S (2023). Botrytis hypersensitive response inducing protein 1 triggers noncanonical PTI to induce plant cell death. Plant Physiol.

[105] Lim PO, Kim HJ, Nam HG (2007). Leaf Senescence. Annu Rev Plant Biol.

[106] Etalo DW, Stulemeijer IJE, Peter van Esse H, de Vos RCH, Bouwmeester HJ, Joosten MHAJ (2013). System-wide hypersensitive response-associated transcriptome and metabolome reprogramming in tomato1[W][OA]. Plant Physiol..

[107] Ma X, Zhang Y, Turečková V, Xue G-P, Fernie AR, Mueller-Roeber B (2018). The NAC transcription factor SlNAP2 regulates leaf senescence and fruit yield in tomato1[OPEN]. Plant Physiol.

[108] Zhang D, Zhu Z, Gao J, Zhou X, Zhu S, Wang X (2021). The NPR1-WRKY46-WRKY6 signaling cascade mediates probenazole/salicylic acid-elicited leaf senescence in Arabidopsis thaliana. J Integr Plant Biol.

[109] Miao Y, Zentgraf U (2007). The antagonist function of arabidopsis WRKY53 and ESR/ESP in leaf senescence is modulated by the jasmonic and salicylic acid equilibrium. Plant Cell.

[110] Rivas-San Vicente M, Plasencia J (2011). Salicylic acid beyond defence: its role in plant growth and development. J Exp Bot.

[111] Radojičić A, Li X, Zhang Y (2018). Salicylic acid: a double-edged sword for programed cell death in plants. Front Plant Sci.

[112] Guo Y, Ren G, Zhang K, Li Z, Miao Y, Guo H (2021). Leaf senescence: progression, regulation, and application. Mol Hortic.

[113] Li R, Li Y, Zhang Y, Sheng J, Zhu H, Shen L (2021). Transcriptome analysis reveals that *SlNPR1* mediates tomato fruit resistance against *Botrytis cinerea* by modulating phenylpropanoid metabolism and balancing ROS homeostasis. Postharvest Biol Technol.

[114] Lee S, Ishiga Y, Clermont K, Mysore KS (2013). Coronatine inhibits stomatal closure and delays hypersensitive response cell death induced by nonhost bacterial pathogens. PeerJ.

[115] Li M, Si X, Liu Y, Liu Y, Cheng X, Dai Z (2022). Transcriptomic analysis of ncRNA and mRNA interactions during leaf senescence in tomato. Int J Biol Macromol.

[116] Caarls L, Elberse J, Awwanah M, Ludwig NR, de Vries M, Zeilmaker T (2017). Arabidopsis JASMONATE-INDUCED OXYGENASES down-regulate plant immunity by hydroxylation and inactivation of the hormone jasmonic acid. Proc Natl Acad Sci U S A.

[117] Huang P-Y, Catinot J, Zimmerli L (2016). Ethylene response factors in Arabidopsis immunity. J Exp Bot.

[118] Hu C, Wu S, Li J, Dong H, Zhu C, Sun T (2022). Herbivore-induced Ca2+ signals trigger a jasmonate burst by activating ERF16-mediated expression in tomato. New Phytol.

[119] Song C, Cao Y, Dai J, Li G, Manzoor MA, Chen C (2022). The multifaceted roles of MYC2 in plants: toward transcriptional reprogramming and stress tolerance by Jasmonate signaling. Front Plant Sci.

[120] Thines B, Katsir L, Melotto M, Niu Y, Mandaokar A, Liu G (2007). JAZ repressor proteins are targets of the SCFCOI1 complex during jasmonate signalling. Nature.

[121] Zhai Q, Deng L, Li C (2020). Mediator subunit MED25: at the nexus of jasmonate signaling. Curr Opin Plant Biol.

[122] Hu C, Wei C, Ma Q, Dong H, Shi K, Zhou Y (2021). Ethylene response factors 15 and 16 trigger jasmonate biosynthesis in tomato during herbivore resistance. Plant Physiol.

