# Supplementary material for: Network analyses predict major regulators of resistance to early blight disease complex in tomato
Source: BMC Plant Biol. 2024 Jul 6;24:641. doi: 10.1186/s12870-024-05366-0 (PMC11227178; doi:10.1186/s12870-024-05366-0)
Supplement: Supplementary file 1 — Supplementary Material 1. [file 12870_2024_5366_MOESM1_ESM.pdf]

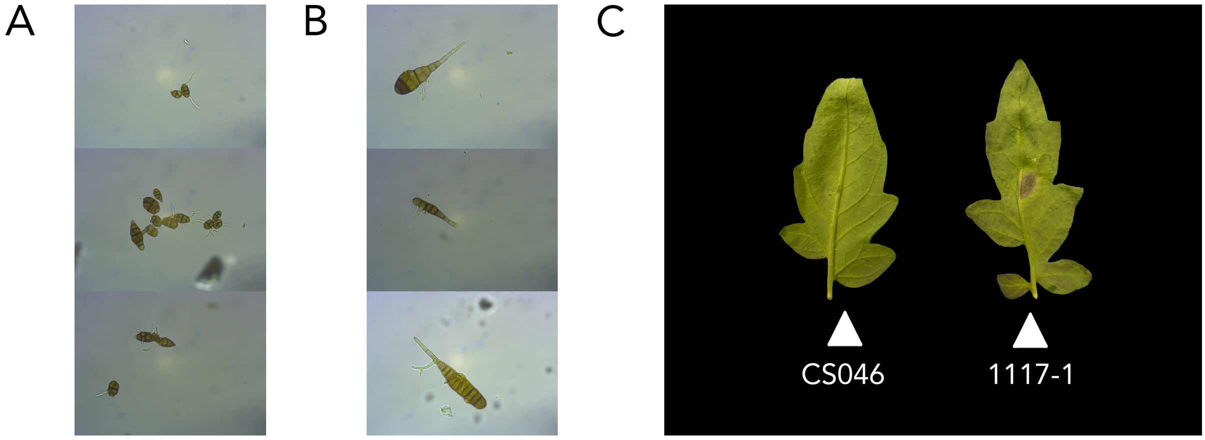

Figure S1. Conidia of CS046 and 1117-1 both readily germinate within 3 hours post infection on Heinz 1706 tomato leaf surface, but only 1117-1 is virulent
(A-B) Representative images captured with a light microscope at 400X magnification of germinating conidia of (A) CS046 and (B) 1117-1, and (C) macroscopic view of detached leaves treated with a 10μL drop infection of 30,000 conidia per mL 72 hours post infection.
